# G protein-coupled receptor signaling in osteogenic bone mesenchymal stem/stromal cells

**DOI:** 10.3389/fcell.2026.1795445

**Published:** 2026-06-01

**Authors:** Trevor J. Wolfe, Samantha R. Weaver

**Affiliations:** Department of Comparative Biosciences, University of Wisconsin-Madison, Madison, WI, United States

**Keywords:** anabolic therapeutics, bone mesenchymal stem/stromal cell, G protein-coupled receptor, GPCR effector, osteoporosis

## Abstract

Skeletal integrity is maintained throughout the lifespan by tightly coordinated actions of cells in the bone microenvironment. In healthy bone, matrix resorption by osteoclasts is balanced by new matrix synthesis by osteoblasts. Tipping this balance to favor resorption depletes bone mineral and disrupts microarchitecture, eventually leading to osteoporosis. Bone mesenchymal stem/stromal cells (BMSCs) are the progenitors of osteoblasts and play a central role in maintaining bone mass. With age and disease, BMSC number and function decline. Defining mechanisms that restore the mesenchymal progenitor population may yield targets for new osteoporosis therapeutics. G protein-coupled receptors (GPCRs) are a major class of receptors through which systemic hormones, neural inputs, mechanical cues, and local paracrine factors converge to regulate BMSC fate, survival, and differentiation. Although GPCRs are the target of approximately one-third of all drugs, only a small number of GPCR-mediated anabolic therapies are currently available to treat osteoporosis. Here, we synthesize the current understanding of classical GPCR signaling pathways in BMSCs and discuss how their dysregulation contributes to bone loss. We further highlight emerging non-classical GPCR targets and effectors that may work to expand the healthy BMSC pool, thereby enhancing bone formation. Defining druggable GPCR signaling axes in BMSCs is a promising strategy to develop safe and effective anabolic therapeutics for osteoporosis.

## 1 Introduction

Bone remodeling is an intricate, balanced process that is essential for maintaining skeletal health and facilitating repair throughout an individual’s lifetime. G protein-coupled receptors (GPCR) play critical roles in regulating this skeletal turnover (Zhang et al., 2025a). Ubiquitously expressed across various tissue and cell types, GPCRs have long been key targets for drug development; approximately 33% of all currently marketed drugs work through GPCRs (Hauser et al., 2017; Zhang et al., 2024; Zhang et al., 2025a). In the musculoskeletal system, GPCRs modulate the functions of numerous and varied cell types, including bone mesenchymal stem/stromal cells (BMSCs), osteoblasts, osteoclasts, and chondrocytes, regulating processes that span cell survival, metabolism, motility, and communication (Zhang et al., 2025b).

Osteoporosis is a prevalent and chronic disease characterized by low bone mass and destruction of microarchitecture, leading to skeletal fragility and enhanced fracture risk. To maintain healthy bone, resorption by myeloid-derived osteoclasts must be in constant balance with new matrix formation by mesenchymal-lineage osteoblasts. Diminishing BMSC number and function play significant roles in tipping this balance, permitting uncoupled mineral loss and depleting bone mineral density (Kiernan et al., 2017). BMSCs are multilineage cells which give rise to osteoblasts, adipocytes, and chondrocytes, have capacity for self-renewal, and play varied roles throughout the skeletal lifespan (Aghebati-Maleki et al., 2019; Zhang W. et al., 2025). With aging and osteoporosis, BMSCs accumulate DNA damage to induce senescence, undergo apoptosis, lose osteogenic capacity, and have altered mechanical and hormonal responsiveness. Expanding the healthy BMSC pool is a promising strategy for development of novel anabolic osteoporosis therapies (Pignolo et al., 2021).

Compared with the large number of antiresorptive agents approved by the FDA for treatment of osteoporosis, there are only three drugs with anabolic properties: teriparatide, abaloparatide, and romosozumab. Teriparatide and abaloparatide activate the parathyroid hormone receptor (PTH1R), while romosozumab is a monoclonal antibody that inhibits sclerostin, enhancing Wnt signaling. While these anabolic therapies are essential tools for patients at high fracture risk, their overall clinical use is much less common than antiresorptives. New GPCR targets are now emerging that show promise for the development of safe and effective therapeutics for osteoporosis. While there are numerous, excellent reviews on GPCR signaling in bone biology and disease (Luo et al., 2019; Zhang et al.., 2025a), as well as reviews on the role of BMSCs in osteoporosis pathogenesis (Hu et al., 2018; Kiernan et al., 2017; Wang et al., 2025a), this review seeks to harmonize these topics. Herein, we highlight the classical GPCR signaling cascades in BMSCs, summarize currently available GPCR-mediated anabolic therapeutics, and describe new GPCRs and GPCR effectors that drive anabolism in BMSCs and bone tissue.

## 2 Osteoporosis

Osteoporosis is a skeletal disorder characterized by low bone mineral density (BMD) and deteriorated bone microstructure, both of which predispose osteoporotic individuals to low-impact fragility fractures (Hernlund et al., 2013). Primary osteoporosis develops from aging and declining sex hormones, while secondary osteoporosis results from immobilization (e.g., after surgery), disease (e.g., cancer), drugs (e.g., glucocorticoids), or other age- and hormone-independent factors (Lehmann et al., 2025). At the cellular level, osteoporosis is essentially a failure of the skeleton to regenerate at the same pace and magnitude at which it is lost, although the mechanisms driving this disbalance are multifaceted (Manolagas, 2000).

While most common in postmenopausal women, osteoporosis also affects a significant proportion of elderly men. Globally, an estimated 1 in 3 women and 1 in 5 men over the age of 50 will experience an osteoporotic fracture in their lifetime (Morin et al., 2025). Such fractures result in 1-year mortality rates of approximately 33% in elderly patients in addition to increased morbidity and disability (Compston et al., 2019; Nikitovic et al., 2013; Walker and Shane, 2023). Individuals who experience osteoporotic fractures also have a greater risk of subsequent fractures (Williamson et al., 2017). While most fragility fractures occur in the vertebrae and proximal femora, other common sites include the ribs, pelvis, proximal humerus, distal radius, and calcaneus (Nuti et al., 2019). The functional consequences of osteoporotic fractures are severe, with approximately 20%–30% of patients failing to regain pre-injury functional status (Yu and Xia, 2019). Treatment is complicated by the fact that patients typically do not present any observable symptoms and may be unaware of their low bone mass (Lehmann et al., 2025). Although historically considered a disease only affecting postmenopausal women and elderly men, younger adults also experience osteoporosis and fracture. These patients merit special attention, as the pathophysiology, diagnostic criteria, and optimal management of osteoporosis in younger adults is far less understood (Herath et al., 2022).

The population-level burden of osteoporotic fracture continues to rise worldwide. Between 1990 and 2019, the global incidence, prevalence, and years lived with disability (YLD) of hip fracture in patients aged 55 years and older increased by 160%, 163%, and 113%, respectively (Feng et al., 2024). During the same period, osteoporosis-related deaths and disability-related life years lost increased by 111% and 94% (Shen et al., 2022). This trajectory is expected to persist, with the annual number of hip fractures projected to nearly double by 2050 (Sing et al., 2023), creating substantial economic strain. In the United States, the average total healthcare cost per osteoporotic fracture exceeds $30,000 per patient, of which approximately $3,000 is paid out of pocket (Williams et al., 2020).

Age-related bone loss is the primary driver of changes in bone mass and microarchitecture, with reductions in volumetric bone mineral density (BMD) detectable as early as the third decade of life in both men and women (Drake et al., 2015). Lifetime spinal trabecular bone loss is substantial; approximately 45% in men and 55% in women (Riggs et al., 2004). In contrast, cortical bone loss typically begins at midlife. From age 50 onward, cortical bone declines by approximately 18% in men and 28% in women (Riggs et al., 2004; Drake et al., 2015). In women, accelerated bone loss is largely driven by menopause-associated estrogen deficiency. During this period, both bone resorption and formation increase, but resorption at roughly twice the rate of formation (Garnero et al., 1996). Estrogen loss increases receptor activator of nuclear factor-κB ligand (RANKL), reduces osteoprotegerin (OPG) expression, and prolongs osteoclast survival by suppressing apoptosis (Eghbali-Fatourechi et al., 2003; Hofbauer et al., 1999; Shevde et al., 2000; Srivastava et al., 2001). Declining estrogen is accompanied by rising follicle-stimulating hormone (FSH), which correlates closely with hip and spine BMD loss in perimenopausal women (Ebeling et al., 1996; Sowers et al., 2006). While testosterone is the primary sex steroid for men, data from cross-sectional (Amin et al., 2000; Center et al., 1999; Greendale et al., 1997; Slemenda et al., 1997) and longitudinal (Khosla et al., 2001) studies demonstrate that BMD correlates more strongly with bioavailable estradiol than with testosterone. In older men, estradiol levels below ∼15 pg/mL are associated with cortical and trabecular bone loss (Khosla et al., 2005).

**FIGURE 1:**
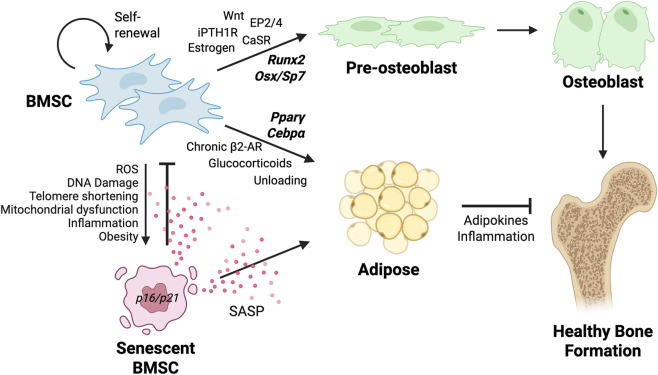
Regulation of BMSC fate decisions. In healthy bone, a self-renewing pool of BMSCs favors osteogenic fate differentiation, stimulating osteoblastogenesis over adipogenesis. Skewing towards the osteogenic lineage occurs through activation of *Runx2* and *Osx*/*Sp7* transcription factors, producing pre-osteoblasts and osteoblasts that support bone formation. Pro-osteogenic signaling inputs include systemic estrogen, Wnt/β-catenin, intermittent PTH (iPTH1R), EP2/4, and CaSR agonism. In contrast, activation of adipogenic transcriptional regulators *Pparg* and *Cebpa*, driven by chronic β2-adrenergic signaling, lack of mechanical input, and glucocorticoids promotes differentiation into bone marrow adipocytes. Cellular stressors drive BMSC senescence and acquisition of a senescence-associated secretory phenotype (SASP), which further biases linage allocation towards adipogenesis. Adipocyte-derived adipokines and inflammatory signals suppress osteoblast function, inducing low bone mass that underlies osteopososis.

BMSC dysfunction plays a central role in the development of osteoporosis (Figure 1). During osteoporosis pathogenesis, there is reduced BMSC commitment to the osteoblast lineage, instead shifting cell fate towards adipogenesis (Li et al., 2015a; Moerman et al., 2004; Nehlin et al., 2019; James, 2013). This shift is regulated by numerous variables, including systemic factors (e.g., glucocorticoids, estrogen), mechanical inputs, expression of key transcription factors, microRNAs, epigenetic regulators, and disrupted signaling cascades (Hu et al., 2018; Fan et al., 2017; Wang B. et al., 2025; Bensreti et al., 2023; Syed et al., 2008; Pierce et al., 2019; Zhang et al., 2021; Xu F. et al., 2021). The BMSC pool also diminishes with age and disease, and those cells that remain are more likely to be inflammatory and/or senescent, with impaired self-renewal capacity and enhanced adipogenic skewing (Pignolo et al., 2021; Zhang et al., 2025a; Cassidy et al., 2024; Bonyadi et al., 2003). Despite these challenges, BMSCs remain an attractive option for clinical application due to their ability to self-renew, ease of culture and expansion *ex vivo*, and suitability for gene therapy. While animal studies have shown promise in using BMSCs to delay or treat osteoporosis, their translation to human patients remains experimental and limited (Aghebati-Maleki et al., 2019; Huang M. et al., 2025). Poor *in vivo* survival of transplanted BMSCs, immunogenicity, tumorigenicity, and regulatory concerns represent a few of the many barriers limiting application of BMSCs for bone regeneration (Huang W. et al., 2025). Research into the basic mechanisms underlying BMSC survival, osteogenic potential, and longevity has enabled the development of bone anabolic therapies and continues to reveal novel targets for drug discovery.

## 3 G protein-coupled receptors (GPCRs)

GPCRs are the most abundant transmembrane protein family implicated in numerous biological processes, including bone development and remodeling (Luo et al., 2019), neurotransmitter signaling (Grammatopoulos, 2017), inflammation and immune response (Lämmermann and Kastenmüller, 2019), homeostasis maintenance (Hazell et al., 2012), and tumor growth and metastasis (Arora et al., 2024; Chaudhary and Kim, 2021) among others. They have easily targeted ligand-binding domains and interact with diverse chemical modulators (Luo et al., 2019). As such, GPCRs account for 12% of all human protein drug targets; approximately 34% of FDA-approved drugs induce therapeutic effects through GPCR signaling (Hauser et al., 2017; Santos et al., 2017). To date, over 800 GPCRs with common structural motifs have been identified (Luo et al., 2019).

### 3.1 Classification

The most frequently used method of classifying GPCRs is to designate classes by letters A-F, with grouping based on amino acid sequences and functional similarities (Attwood and Findlay, 1994; Kolakowski, 1994). Class A (i.e., rhodopsin-like family) is the largest group, accounting for over 80% of GPCRs. Members of this class include hormone, neurotransmitter, and light receptors, and are structurally defined by eight total transmembrane (TM) helices and a palmitoylated cysteine residue near the C terminus. Class B, or the secretin receptor family, consists of around 70 receptors that are characterized by a long N-terminal domain stabilized by disulfide bonds. The metabotropic glutamate family, GABA receptors, calcium sensing receptors (CaSR), and taste receptors all belong to class C and share a distinct ∼600 residue-long N-terminal ligand binding domain. Finally, classes D, E, and F include fungal mating pheromone receptors, cAMP receptors, and frizzled/smoothened receptors respectively (Hu et al., 2017).

Several groups within the A-F system have no human members, and there are GPCRs that do not fit into any of the groups (Luo et al., 2019). Accordingly, an alternative classification system, called GRAFS, groups GPCRs into five families: glutamate (G), rhodopsin (R), adhesion (A), frizzled/taste2 (F), and secretin (S) (Schiöth and Fredriksson, 2005). The primary difference between each system is the division of class B into separate secretin and adhesion families based on their distinct evolutionary histories (Hu et al., 2017). Finally, roughly 150 putative human GPCRs with unknown functions and ligands are designated as orphan receptors.

### 3.2 Signaling principles

GPCR signaling is initiated upon receptor activation by extracellular ligands, such as ions, amines, nucleotides, peptides, proteins, lipids, organic odorants, and photons (Schiöth and Fredriksson, 2005). The ensuing signaling cascade is then mediated by GPCR effectors, including heterotrimeric G proteins, GPCR kinases (GRKs), and arrestins (Kobilka, 2007; Lefkowitz, 1998). Heterotrimeric G proteins contain α, β, and γ subunits and are key GPCR signaling transducers (Hurowitz et al., 2000; Oldham and Hamm, 2008). Gα subunits are designated into four main classes according to their sequence: Gαs, Gαi/o, Gαq, and Gα13 (McCudden et al., 2005; Milligan and Rees, 1999). GPCR activation induces a conformational shift in the bound G protein, causing the Gα subunit to exchange GDP for GTP and subsequently dissociate from the Gβγ dimer (Luo et al., 2019). Free GTP-bound Gα and the Gβγ dimer then activate effectors for downstream signaling, such as adenylyl cyclase (AC), potassium channels, or phospholipase (Riddle et al., 2005; Wieland et al., 2007). Importantly, GPCR signaling can be either stimulatory or inhibitory. Gαs stimulates AC-mediated production of cAMP and Gαq activates the phospholipase C pathway (Harden et al., 2011), while Gαi/o inhibits cAMP production (Serezani et al., 2008). GPCR signaling is terminated via GRK-mediated phosphorylation of the intracellular loops and C-terminal tail of the receptor (Luo et al., 2019). Subsequent recruitment of arrestins excludes G protein interaction and induces receptor-arrestin complex internalization, terminating signal transduction (Pitcher et al., 1998; Premont and Gainetdinov, 2007).

### 3.3 Relevance to bone biology

More than 56 GPCRs have been identified as key regulators of skeletal homeostasis (Zhang et al., 2025a). These receptors orchestrate specialized functions within cells of the bone microenvironment, including BMSCs, osteoblasts, osteoclasts, osteocytes, chondrocytes, and immune cells (Luo et al., 2019; Marino and Bellido, 2024; Wang et al., 2021). Aberrant GPCR signaling induces musculoskeletal dysfunction and disease (Zhang et al., 2025a). Monogenic GPCR-associated bone diseases include Jansen metaphyseal chondrodysplasia (gain-of-function PTH1R), Blomstrand chondrodysplasia (loss-of-function PTH1R), autosomal dominant hypocalcemia type I (gain-of-function in CaSR), and familial hypocalciuric hypercalcemia (loss-of-function CaSR) (Hannan et al., 2016; Portales-Castillo et al., 2025). Although these disorders are rare, they highlight the central role of GPCR pathways in skeletal biology and suggest that more common genetic variation within these signaling networks may also influence bone mass.

As BMD is heritable, genome-wide association studies (GWAS) have sought to define the genetics of bone mass in human populations (Boudin et al., 2016). Numerous single nucleotide polymorphisms (SNPs) have been associated with osteoporosis and BMSC function across human populations (Table 1) (Sopova et al., 2025b; Luo et al., 2019). Recently, a combination of three SNPs in GPCR genes (*FSHR, TSHR, ADRB2*) was identified with a frequency of approximately 20% in a cohort of women with postmenopausal osteoporosis. Follow up in patient-specific BMSCs *in vitro* confirmed impaired osteogenic differentiation and mineralization (Sopova et al., 2025b; Sopova et al., 2025a). This highly translational strategy not only defines specific targets for novel therapeutics but also facilitates genetic screening and risk stratification to inform monitoring strategies (Forgetta et al., 2020; Wang et al., 2026). As the FDA recently approved BMD as a qualified endpoint in clinical trials for osteoporosis drugs instead of relying solely on fracture incidence, there is an opportunity for development of mechanistically targeted therapies that directly modulate GPCR-drive anabolic pathways in skeletal cells (Black et al., 2024).

**TABLE 1:** GPCR genetic variants associated with osteoporosis and BMSC function in humans.

GPCR name	Class	SNP(s)*	Functional studies in human BMSCs
ADRB2	A	rs1042713	Hedderich et al. (2020), Krasnova et al. (2024), Sopova et al. (2025b)
CASR	C	rs1801725	Cho et al. (2020), Pipino et al. (2014), Sarem et al. (2018)
CB2	A	rs2501431 rs3003336 rs2229579 rs4237	Wang B. et al. (2018), Tian et al. (2021)
DRD2	A	rs1800497	Wang et al. (2020)
FSHR	A	rs6166	Ning et al. (2025), Sopova et al. (2025b)
FZD1	​	rs2232157 rs2232158	Yu et al. (2015)
P2YR2	A	rs2511241	Zippel et al. (2012), Ciciarello et al. (2013), Noronha-Matos et al. (2012)
PTH1R	B	rs1138518	Sammons et al. (2004), Pountos et al. (2010), Kulebyakin et al. (2022)
RXFP2	A	rs121918303	Escobar et al. (2023)
TSHR	A	rs1991517	Bagriacik et al. (2012), Sopova et al. (2025b)

* SNPs, and associated references compiled by (Sopova et al., 2025b; Luo et al., 2019).

## 4 Classical GPCR signaling in bone mesenchymal stem/stromal cells (BMSCs)

### 4.1 Gs-coupled GPCRs

The Gαs-AC-cAMP-PKA pathway is a fundamental signal transduction cascade that generally promotes osteogenesis, inhibits adipogenesis, and induces BMSC survival. When Gαs-coupled GPCRs are bound by a ligand, the Gαs protein activates membrane-bound AC, triggering accumulation of cAMP and activation of PKA to drive a variety of downstream cellular responses. Elevating basal Gαs activity in murine osteoblasts promotes age-dependent increases in trabecular bone, with particularly high responsiveness in the early postnatal period (Abo-Elenin et al., 2025; Hsiao et al., 2008; Hsiao et al., 2010a; Hsiao et al., 2010b; Wattanachanya et al., 2015), although the bone may not be of high quality (Zhang et al., 2017). In human BMSCs, knockdown of the gene locus that encodes for the alpha subunit of the Gαs protein (*GNAS*) promotes aberrant osteogenic differentiation, contributing to premature fusion of cranial sutures (Yan et al., 2025).

**FIGURE 2:**
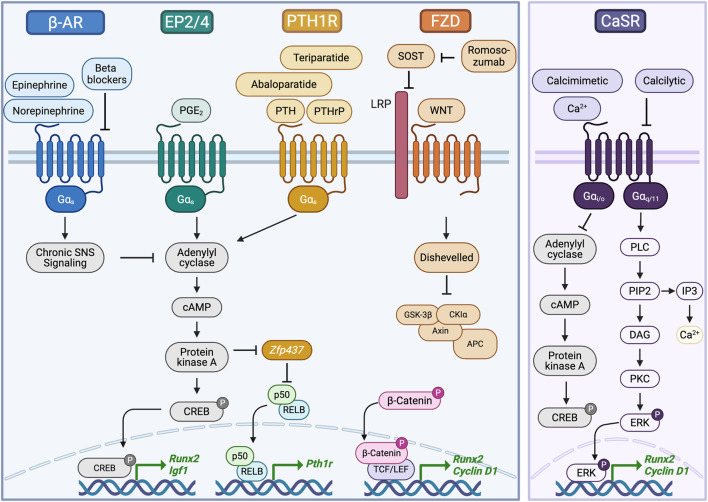
Major anabolic GPCR signaling cascades in BMSCs. Current bone anabolic compounds target Gαs-, Gαi/o-, and Gαq/11-coupled GPCRs. Upon activation by their respective ligands and/or anabolic agent, β-AR, EP2/4, and PTH1R induce cAMP production and CREB phosphorylation to ultimately promote the expression of osteogenic commitment genes. Persistent SNS signaling through β-AR elevates cAMP but paradoxically suppresses osteogenesis, an effect that can be mitigated by beta blockers. Many Gαs signaling pathways also enhance osteogenesis through crosstalk with Wnt/β-catenin signaling, including inhibition of GSK3β and subsequent β-catenin nuclear accumulation. Calcimimetics and calcilytics modulate CaSR, which couples to Gαi/o and Gαq/11 G-proteins. G⍺i/o-coupled CaSR signaling inhibits the canonical cAMP pathway, while Gαq/11 signaling stimulates the PLC/IP3/DAG and ERK pathways, ultimately inducing the expression of osteogenic commitment genes.

There are several classical Gαs-coupled GPCRs that play essential roles in BMSC differentiation and function (Table 2; Figure 2) (Liu et al., 2024). Canonical Gαs-PTH1R signaling induces BMSC proliferation and survival, osteoblast lineage commitment, and osteogenic gene expression (Chen et al., 2016). Simultaneously, intermittent PTH1R agonism inhibits adipogenic differentiation of BMSCs (Chen et al., 2021; Fan et al., 2017). More recent work has shown that zinc finger protein 467 (*Zfp467*) negatively regulates PTH1R expression, as deletion of *Zfp467* induces BMSC osteogenesis and high bone mass (Liu et al., 2023). Intermittent PTH injections were the first anabolic agents approved for osteoporosis therapy in the United States and are discussed in detail in Section 5.

**TABLE 2:** Major and emerging GPCRs regulating BMSC osteogenesis.

GPCR name	Class	Primary coupled G-protein	Signaling pathway	References relevant to BMSCs
ADORA2A/B	A	G⍺_s_	cAMP/PKA	Carroll et al. (2012), Katebi et al. (2009), Park et al. (2024), Zheng and Wang (2020)
β-AR	A	G⍺_s_	cAMP/PKA	Hedderich et al. (2020), Sopova et al. (2025b)
CaSR	C	G⍺_q/11_ and G⍺_i/o_	MAPK/Akt	Cho et al. (2020), González-Vázquez et al. (2014), Gowen et al. (2000), Liu et al. (2025), Rybchyn et al. (2019), Xu et al. (2012)
CB1/2	A	G⍺_i/o_	MAPK/Akt	Ofek et al. (2006), Samir and Malek (2014), Sophocleous et al. (2011), Tian et al. (2021), Wang B. et al. (2018)
EP2/4	A	G⍺_s_	cAMP/PKA	(Chen et al., 2019; Li et al., 2005; Paralkar et al., 2003; Shamir et al., 2004; Weinreb et al., 2006; Xi et al., 2025)
FZD	F	-	Wnt/β-catenin	(Yu et al., 2015; Hang et al., 2019; Yu et al., 2024; Wang et al., 2025a)
GLP-1R	B	G⍺_s_	cAMP/PKA	(Abo-Elenin et al., 2025; Cheng et al., 2022; Lu et al., 2015; Ma et al., 2013; Nuche-Berenguer et al., 2009; Nuche-Berenguer et al., 2010; Nuche-Berenguer et al., 2011; Wang et al., 2017; Sun et al., 2015; Tian et al., 2025; Yang et al., 2019)
GPR81	A	G⍺_i/o_	Wnt/β-catenin	Guo et al. (2025)
GPR120	A	G⍺_q/11_	MAPK	Ahn et al. (2016)
GPR126	Adhesion	G⍺_s_	cAMP/PKA	(Liebscher et al., 2014; Sun et al., 2020)
GPR133	Adhesion	G⍺_s_	cAMP/PKA	Lehmann et al. (2025)
KISS1R	A	G⍺_q/11_	PLC/PKC	(Comninos et al., 2022; Son et al., 2018)
LGR4/5/6	A	G⍺_s_	cAMP/PKA	(Doherty et al., 2023; Khedgikar et al., 2022; Khedgikar and Lehoczky, 2019; King et al., 2024; Luo et al., 2009; Sun et al., 2019)
LPAR1/4	A	Multiple	RhoA/ROCK1/β-catenin	(Liu et al., 2010; Xie et al., 2020b; Park et al., 2024)
NPFFR1/2	A	G⍺_i/o_	cAMP/PKA	Yu et al. (2023)
NPY1R	A	G⍺_i/o_	MAPK/Akt	(Yu et al., 2016; Liu et al., 2016; Xie et al., 2020a)
PTH1R	B	G⍺_s_	cAMP/PKA	(Balani et al., 2017; Fu et al., 2025; Tang et al., 2019; Yu et al., 2012; Chen et al., 2016; Chen et al., 2021; Fan et al., 2017; Liu et al., 2023; D'Amelio et al., 2012; Cohen et al., 2017; Kuo et al., 2017; Xiao et al., 2026; Lv et al., 2020)
TGR5	A	G⍺_s_	AMPK	Wang Q. et al. (2018)

Binding of epinephrine or norepinephrine to the Gαs-coupled β-adrenergic receptors inhibits mouse- and human-derived BMSC proliferation and osteogenesis *in vitro* (Hedderich et al., 2020; Li et al., 2010). When activated by the sympathetic nervous system, β-adrenergic receptors inhibit BMSC osteogenesis by preventing Gαs-AC-cAMP-PKA signaling. The inhibitory effects of persistent β-adrenergic signaling on BMSC osteogenesis are reversible upon treatment with beta-blockers, such as propranolol (Zhong and Xia, 2021). These data suggested that patients taking beta blockers may have improved skeletal health due to adrenergic receptor antagonism. While both the β1 and β2 receptors are detectable in human bone, only patients treated with β1-selective blockers show improved bone mass in a randomized clinical trial. Enhanced BMD in these patients is mediated by reduced bone resorption rather than increased bone formation (Khosla et al., 2018). *In vitro*, culturing mouse BMSCs with intermittent PTH in conjunction with the beta blocker propranolol had a greater osteoblastogenesis effect than administration of PTH alone, translating to osetoblastogenesis and high bone mass in mice *in vivo* (Huang et al., 2024). Combination therapy of beta blockade with intermittent PTH therapy to enhance bone formation merits further clinical exploration.

Finally, Prostaglandin E2 (PGE2) binding to its Gαs-coupled receptors EP2 and EP4 stimulates BMSC function and bone anabolism (Shamir et al., 2004; Weinreb et al., 2006). Germline deletion of EP4 induces osteopenia and delays fracture healing in mice (Li et al., 2005), while administration of an EP2 selective agonist induces fracture healing (Paralkar et al., 2003). More recently, the PGE2/EP4 axis has been characterized as a critical neurosensory axis regulating bone mass. Osteoblast-derived PGE2 activates EP4 in sensory nerves to induce bone formation, with injection of small-molecule PGE2 agonists enhancing bone mass in rodent models (Chen et al., 2019). In another murine model, administration of the traditional Chinese herbal formula Jinkui Shenqi Pill similarly elevated serum PGE2, enhanced BMSC and neuronal expression of EP4, and stimulated fracture healing *in vivo* (Xi et al., 2025). While there is some interest in exploring the PGE2/EP2-4 axis for therapeutic treatment of osteoarthritis, less effort has been made towards leveraging PGE2 signaling for bone anabolism (Zhang et al., 2025b).

### 4.2 Gαi/o-coupled GPCRs

Gαi/o coupled GPCR signaling cascades also regulate BMSC differentiation and survival (Figure 2). Upon ligand binding to Gαi/o-coupled GPCRs, a conformational change in the receptor causes G⍺i/o-βγ subunit dissociation. The activated G⍺i/o subunit inhibits AC, reducing intracellular cAMP levels, while the Gβγ subunit activates other modulators, such as G protein-gated inwardly rectifying K^+^ (GIRK) channels. Constitutive blockade of receptor-activated Gαi/o signaling in osteoblasts induces site, age, and sex-specific increases in bone formation and accelerates fracture healing (Millard et al., 2011; Millard et al., 2017; Wang et al., 2015). Suppressing Gαi/o signaling in osteoblasts also enhances the effects of intermittent PTH *in vivo*, stimulating both trabecular and cortical bone formation in a rodent model (Wang et al., 2025b).

C-X-C chemokine receptor type 4 (CXCR4) is a class A GPCR bound by the chemokine CXCL12 (i.e., stromal derived factor 1; SDF-1). In BMSCs, Gαi/o-coupled CXCR4 signals through BMP2, PI3K/AKT, and ERK/MAPK to induce β-catenin stabilization, proliferation, survival, and transcription of osteogenic genes (Guang et al., 2013; Xiong et al., 2021; Shahnazari et al., 2013; Zhu et al., 2011; Tzeng et al., 2018). Inhibition of CXCR4 signaling prevents osteogenic differentiation of human-derived BMSCs (Hosogane et al., 2010; Larraz-Prieto et al., 2025). However, unbalanced CXCR4 activation is also detrimental to osteogenesis, as patients with a rare gain-of-function CXCR4 mutation (WHIM Syndrome), have low bone mass, with poor mineralization of primary BMSC cultures *in vitro* (Anginot et al., 2023). CXCR4 is a target of regenerative strategies for fracture repair due to its prominent role as a chemotactic signal for BMSC recruitment (Xie et al., 2026; Zhang et al., 2022; Liu et al., 2020).

Unlike CXCR4, the CaSR has been targeted as a putative therapeutic target for osteoporosis. Activation of CaSR by extracellular Ca^2+^ enhances BMSC proliferation and differentiation (González-Vázquez et al., 2014; Cho et al., 2020; Xu et al., 2012). CaSR signaling also promotes recruitment of BMSCs at sites of active remodeling, further enhancing osteogenesis (Cianferotti et al., 2015). Class C GPCR signaling from CaSR is mediated by both Gαi/o and Gαq/11 G-proteins (Figure 2). Recent work has implicated the scaffolding protein Homer1 as a key mediator of CaSR-mediated activation of AKT and Wnt (Rybchyn et al., 2019; Liu et al., 2025). Due to its essential role in maintaining normocalcemia, perturbations in CaSR have significant physiological consequences. As is exemplified by CaSR, designing novel GPCR-based anabolic bone therapeutics is complex and intricate: activation of osteolineage cells must not be at the expense of larger mineral or systemic effects.

## 5 Current GPCR-mediated anabolic therapeutics

**FIGURE 3:**
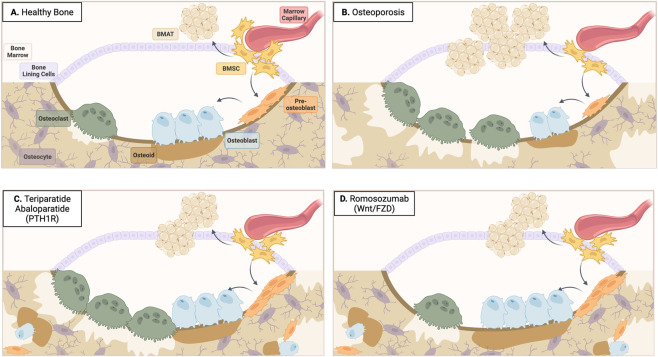
Effect of anabolic drugs on the bone remodeling unit. Under physiologic conditions **(A)**, bone remodeling is balanced through coordinated activity of BMSCs, osteoblasts, osteoclasts, and osteocytes. BMSCs within the marrow niche proliferate and differentiate into pre-osteoblasts and osteoblasts, yielding osteoid deposition and maintenance of skeletal integrity. During osteoporosis **(B)**, remodeling is uncoupled, with increased osteoclast-mediated resorption and insufficient osteoblast-mediated formation. This imbalance is accompanied by impaired BMSC osteogenic commitment and elevated marrow adiposity, leading to net bone loss. Intermittent activation of PTH1R with teriparatide or abalopartide **(C)** stimulates lineage expansion from BMSCs, enhances osteoblast activity, and promotes new bone formation, with a concomitant increase in bone resorption. Neutralization of osteocyte-derived sclerostin with Romosozumab **(D)** enhances Wnt/β-catenin signaling through FZD-LRP5/6 complexes, promoting BMSC osteogenic differentiation and osteoblast function while also suppressing osteoclast activity, robustly supporting bone mass gain. Abbreviations: BMSC, bone mesenchymal stem/stromal cell; BMAT, bone marrow adipose tissue; FZD, Frizzled receptor; PTH1R, parathyroid hormone receptor 1.

Currently-approved osteoporosis therapeutics fall under one of two categories: those that inhibit bone resorption or those that promote the synthesis of new bone tissue. Antiresorptive treatments have been extensively reviewed elsewhere (Chen and Sambrook, 2011; Reid and Billington, 2022). Far fewer anabolic agents have received FDA approval for osteoporosis, and include teriparatide, abaloparatide, and romosozumab. Of these, teriparatide and abaloparatide target PTH1R to exert their pro-osteogenic effects, while romosozumab enhances canonical Wnt signaling (Figure 3). Calcilytics that mediate CaSR, while originally promising, ultimately failed to receive FDA approval for osteoporosis.

### 5.1 Calcimimetics and calcilytics

A calcimimetic is defined as any ligand that activates CaSR, including agonists (type I) and allosteric activators (type II) (Nemeth and Goodman, 2016; Nemeth, 2013; Widler, 2011). Type I calcimimetics are generally inorganic or organic polycations, such as Mg^2+^ or Gd^3+^, while type II are small natural molecules, synthetic compounds, or peptides (Nemeth and Goodman, 2016). Oral administration of calcimimetics causes a rapid, dose-dependent reduction in plasma levels of PTH and, subsequently, Ca^2+^ (Nemeth and Goodman, 2016). This is achieved by calcimimetic binding to the CaSR to induce conformational changes that enhance sensitivity to extracellular Ca^2+^. By lowering the threshold for CaSR activation, calcimimetics mitigate hypercalcemia-related conditions, including primary and secondary hyperparathyroidism. They have also been utilized experimentally to accelerate fracture healing and treat bone tumors (White and Vilardaga, 2021).

Conversely, calcilytics are CaSR antagonists that act as negative allosteric modulators to rapidly induce intermittent release of PTH into plasma, stimulating bone formation (Nemeth, 2013; Nemeth and Goodman, 2016; Widler, 2011). Ovariectomized rats co-treated with the calcilytic NPS 2143 and 17β-estradiol had elevated BMD compared to rats treated with either drug alone (Gowen et al., 2000). Based on these and other results, three different calcilytics advanced to Phase II clinical trials in women with postmenopausal osteoporosis: ronacalceret (Fitzpatrick et al., 2011), encalceret (Halse et al., 2014), and ATX914 (John et al., 2014). However, all three trials were prematurely terminated due to lack of efficacy, ending clinical development of calcilytics for osteoporosis treatment (Nemeth and Goodman, 2016).

### 5.2 Teriparatide

Teriparatide was the first anabolic therapeutic approved by the FDA in 2002 for treating osteoporosis in postmenopausal women, men with idiopathic or hypogonadal osteoporosis, and women and men with glucocorticoid-induced osteoporosis with high fracture risk (Dede et al., 2017; Lindsay et al., 2016). Teriparatide is an analog of human PTH with an amino acid sequence identical to the 34 N-terminal amino acids (i.e., the biologically active region) of endogenous PTH (Jha, 2023). Intermittent administration of teriparatide enhances bone mass by preventing osteoblast apoptosis and activating Wnt/β-catenin signaling, in part through repression of a glycoprotein secreted primarily by osteocytes that is a potent inhibitor of canonical Wnt/β-catenin signaling, called sclerostin (Wein and Kronenberg, 2018; Wein et al., 2018).

In BMSCs, teriparatide promotes proliferation, survival, osteogenic commitment, and differentiation into osteoblasts (Table 3) (Balani et al., 2017; Fu et al., 2025; Tang et al., 2019; Yu et al., 2012). Postmenopausal women treated with teriparatide have a greater number of more differentiated circulating osteoprogenitors than untreated patients in peripheral blood mononuclear cells (D'Amelio et al., 2012). This pattern is recapitulated in premenopausal women, with teriparatide also increasing osteoprogenitor expression of insulin-like growth factor 1 receptor (IGF-1R) (Cohen et al., 2017). Various molecular regulators have been implicated in the proliferative and anabolic effects of teriparatide on BMSCs, including mTOR, FGF-2, FOXJ3, PKCδ, and miRNAs (Kuo et al., 2017; Xiao et al., 2026; Lv et al., 2020; Fu et al., 2025; Cheng et al., 2019). Collectively, these molecular and cellular effects translate into measurable gains in bone strength, mass, and structural integrity (Brixen et al., 2004; Girotra et al., 2006; Stroup et al., 2008).

**TABLE 3:** Current and emerging GPCR-mediated bone anabolic compounds.

Compound	Primary GPCR target	Mechanism of action and effect in BMSCs
Teriparatide/Abaloparatide	PTH1R	When intermittent, stimulates G⍺s-AC-cAMP-PKA-pCREB and Wnt/β-catenin to induce differentiation, survival, and osteogenic commitment and differentiation while suppressing adipogenesis. Also promotes BMSC survival
Romosozumab	FZD	Monoclonal antibody to prevent Sost-mediated suppression of Wnt signaling, stabilizes β-catenin to induce osteogenic commitment and differentiation while suppressing adipogenesis. Secondary reduction of bone resorption
Calcilytics	CASR	Antagonizes CASR to transiently block G⍺i/o, enabling G⍺s-AC-cAMP-PKA-pCREB. Also induces transient increase of endogenous PTH and activates PTH1R as described in PTH1R box
Ki 16425	LPAR1	Competitively blocks LPA binding to LPAR1, inhibits G⍺i/o-RhoA/ROCK-ERK/MAPK and PI3K/AKT. Contradicting reports on enhancement or suppression of BMSC activity and osteogenesis
ZM 241385	ADORA2A	Competitively blocks adenosine binding to ADORA2A, inhibits G⍺s-AC-cAMP-PKA-pCREB. Contradicting reports on enhancement or suppression of BMSC activity and osteogenesis
AP503	GPR133	Agonizes GPR133, stabilizes active receptor conformation to stimulate G⍺s-AC-cAMP-PKA-pCREB to promote osteogenic commitment and differentiation
HU308	CB2	Agonizes CB2 to stimulate G⍺i/o-MAPK/ERK and PI3K/AKT to promote osteogenic commitment and differentiation. May also suppress adipogenesis and enhance BMSC survival
2-AG	CB1/2	Agonizes CB1 and CB2 to stimulate G⍺i/o-MAPK/ERK and PI3K/AKT to promote osteogenic commitment and differentiation. May also suppress adipogenesis and enhance BMSC survival
Semaglutide	GLP-1R	Agonizes GLP-1R, G⍺s-AC-cAMP-PKA-pCREB to promote BMSC survival and differentiation. Anti-inflammatory effects that may be indirect due to larger systemic effects
GPBARA	TGR5	Agonizes TGR5 to stimulate the AMPK/eNOS pathway, ultimately promoting BMSC osteogenic differentiation and mineralization
Kisspeptin	KISS1R	Agonizes KISS1R, stimulates G⍺q/11 to promote BMSC differentiation into osteoblasts via NFATc4-mediated BMP2 expression
TUG-891	GPR120	Agonizes GPR120; at high concentrations stimulates G⍺q/11 to promote BMSC differentiation into osteoblasts by supporting osteogenic gene transcription

Although the development of teriparatide represents a significant breakthrough in osteoporosis treatment, it is not without its limitations. For one, teriparatide treatment also upregulates cAMP-response element binding protein (CREB)-dependent expression of RANKL, promoting bone resorption in addition to bone formation (Marino and Bellido, 2024). While limited administration (i.e., once per day) of teriparatide preferentially stimulates osteoblast activity, excess PTH1R signaling can induce bone resorption, thus leading to bone loss (Blick et al., 2008; Girotra et al., 2006). Additionally, an increased risk of osteosarcoma with teriparatide was observed during pre-clinical testing on rats, however it is worth noting that this phenomenon has not been observed in humans (Gilsenan et al., 2021; Miller et al., 2021). The total lifetime duration of teriparatide treatment was originally limited to 24 months, however the FDA has recently revised this limitation only for patients who remain at or have returned to a high risk for fracture (Miller et al., 2021). The anabolic effects of teriparatide also wane over extended treatment periods, as evidenced by reduced levels of P1NP, CTX, and osteocalcin, as well as elevated levels of the Wnt inhibitor dickkopf-1 (DKK1), after 12 months (Gatti et al., 2011; Yu et al., 2011).

### 5.3 Abaloparatide

Abaloparatide, a synthetic analogue of human parathyroid hormone-related peptide (hPTHrP), was approved by the FDA in 2017 (Cosman et al., 2022). As with teriparatide, its anabolic effects are mediated through binding to and activation of PTH1R and its subsequent signaling cascade. However, abaloparatide induces less bone resorption and hypercalcemia compared to teriparatide (Tella et al., 2017; Culler et al., 2001; Doyle et al., 2013). Whereas teriparatide preferentially binds the R0 conformation of PTH1R, abaloparatide preferentially binds the RG conformation. As a result, abaloparatide induces more transient PTH1R signaling and therefore a greater net anabolic effect than teriparatide due to a more rapid dissociation from the receptor (Dean et al., 2008; Maeda et al., 2013; Okazaki, 2008).

### 5.4 Romosozumab

Romosozumab is a humanized monoclonal antibody that was FDA approved in 2019 for the treatment of osteoporosis in postmenopausal women at high fracture risk (Fabre et al., 2020; Sølling et al., 2018). Romosozumab exerts its anabolic effects by binding to sclerostin and removing the inhibitory brake on Wnt signaling, permitting Wnt ligands to activate LRP5/6-FZD receptor complexes on BMSCs and osteoblasts (Lim and Bolster, 2017). While canonical Wnt signaling is not GPCR-mediated in the classical sense as there is no requirement for heterotrimeric G proteins, FZD receptors are structurally class F GPCRs. Following Wnt ligand binding, stabilization and nuclear translocation of β-catenin permits transcription of osteogenic target genes, inducing BMSC differentiation, survival, and ultimately osteoblastic matrix production (Table 3). The mechanisms of action of Wnt/β-catenin-mediated BMSC osteogenesis are numerous: Apelin-13, an endogenous ligand for the APJ GPCR, induces Wnt-mediated osteogenesis in human BMSCs *in vitro*, while the herbal compound icariin stimulates human and rat BMSC differentiation via miRNA-mediated activation of Wnt (Hang et al., 2019; Yu et al., 2024; Xu Y. et al., 2021). Efforts have also been made to stabilize β-catenin to induce osteogenic BMSC activity, including GSK3β inhibition, SIRT1 stabilization, and perturbation of Notch signaling (Simic et al., 2013; Hilton et al., 2008; Lee et al., 2021). There is also significant molecular crosstalk between osteocytes, which secrete sclerostin, and BMSCs which has been reviewed elsewhere (Wang et al., 2025a). Despite this rich body of preclinical work, some of which advanced to clinical trials, only sclerostin inhibitors have received FDA approval (Appelman-Dijkstra and Papapoulos, 2018).

Like teriparatide and abaloparatide, romosozumab is not without limitations. Romosozumab is similarly only approved for a 12-month lifetime treatment duration in the United States. The magnitude of romosozumab’s anabolic effect also wanes over time, likely due to compensatory increases in Wnt inhibitors such as DKK1 (Krupa et al., 2024). Recently, promising data demonstrate potential remediation for these challenges, as 3 months of romosozumab followed by 9 months of the bisphosphonate denosumab was equally efficacious for increasing hip BMD compared to 12 months of romosozumab alone in postmenopausal women of high fracture risk (Leder et al., 2026; The Lancet Endocrinology, 2026).

Taken together, the development of teriparatide, abaloparatide, and romosozumab were paradigm shifts in osteoporosis treatment. However, their collective limitations, namely, the need for daily injections, waning efficacy over time, and the limited lifetime dosage duration except in severe cases, necessitates the development of alternative anabolic therapies. Re-examination of the classical PTH1R signaling axis spurred the development of salt inducible kinase (SIK2/SIK3) inhibitors for the treatment of low bone mass, work that is currently ongoing (Sato et al., 2022). Additional new bone anabolic therapies currently in Phase II clinical trials include a dual sclerostin/DKK1 inhibitor (AGA2118), orally available PTH(1-34) (EB613; (Tripto-Shkolnik et al., 2024)), and combination strategies (e.g., combination teriparatide and calcimimetics for osteoporosis in men (Tonk et al., 2022)). Generation of these and other new GPCR-based therapeutics is dependent on a mechanistic understanding of the classical and emerging signaling cascades regulating bone mineral accrual.

## 6 Emerging and non-classical GPCR targets for anabolic therapy

The development of teriparatide, abaloparatide, and romosozumab exemplify how fundamental investigations into GPCR activity can identify targets for translation into effective clinical intervention. New anabolic targets may also arise from characterization of physiologic stimuli known to induce bone mass, such as exercise. For example, high-intensity interval training in ovariectomized mice elevates circulating lactate levels, which promote BMSC osteogenic differentiation through activation of the lactate receptor GPR81 and downstream Wnt signaling (Guo et al., 2025). In parallel, genome wide association studies have identified numerous single nucleotide polymorphisms (SNPs) associated with low bone mass and osteoporosis across human populations (Table 1). Translating these genetic associations into therapeutic strategies requires integration with preclinical and *in vitro* studies to functionally validate emerging GPCR targets that regulate BMSC proliferation and osteogenic differentiation (Table 2).

### 6.1 Class A GPCRs

To identify novel GPCR targets for hard tissue regeneration, repurposing studies of existing GPCR inhibitors revealed a promising subset of Class A GPCRs (Park et al., 2024). Upon evaluating *in vitro* mineralization levels in human dental pulp-derived MSCs (hDSPCs), six GPCRs (LPAR1, F2R, F2RL1, F2RL2, S1PR1, and ADORA2A) as well as their corresponding inhibitors induced osteogenesis. Notably, each of the inhibitors induced greater mineralization compared to tideglusib, a glycogen synthase kinase 3 (GSK3) inhibitor previously reported to stimulate tooth regeneration in animal models (Neves et al., 2017; Park et al., 2024). While all six inhibitors upregulated alkaline phosphatase (ALP) activity and mRNA expression of osteogenic genes in hDSPCs *in vitro*, only Ki 16425 (LPAR1 inhibitor) and ZM 241385 (ADORA2A inhibitor) improved *in vivo* osteogenesis in a rat calvarial defect model (Park et al., 2024).

Other works have also investigated the link between these class A GPCRs and bone homeostasis. LPAR1 antagonism inhibits osteoclast differentiation in mice, preventing bone resorption (David et al., 2014). However, LPAR1 inhibition may also abrogate osteogenesis in human BMSCs (Liu et al., 2010), showing opposite effects of those reported by Park et al. in hDSPCs. Interestingly, other groups have identified a role for another LPAR family member (LPAR4) in BMSCs. Both shRNA- and siRNA-mediated knockdown of LPAR4 enhance osteogenesis in human BMSCs, and LPAR4-deficient mice have increased bone volume fraction, trabecular number, and trabecular thickness (Liu et al., 2010; Xie et al., 2020b). Like LPAR1, F2R and F2RL1 may also regulate resorption alongside formation. F2R overexpression prevents osteoclast formation and function (Zhang et al., 2020) and F2RL1 knockout mice have high bone mass due to reduced resorption, with lower expression of osteogenic genes and compromised BMSC proliferation (Georgy et al., 2012; Sanaei et al., 2021). Finally, while local administration of ZM 241385, the ADORA2A inhibitor, improved bone healing in a rat fracture model (Zheng and Wang, 2020), ADORA2A activation also promotes BMSC proliferation and differentiation (Katebi et al., 2009). Closely-related ADORA2B activation similarly induces BMSC osteogenesis and enhances *in vivo* bone formation (Carroll et al., 2012). These conflicting studies demonstrate the necessity of precise dosing, timing, and careful consideration of preclinical model when examining class A GPCR-mediated osteogenesis.

### 6.2 LGR family members (LGR4/5/6)

Leucine-rich repeat-containing GPCRs (LGRs) also belong to the family of class A GPCRs (Doherty and Sanjay, 2020). LGRs do not induce canonical GPCR signaling. Instead, upon R-spondin ligand binding, LGRs sequester transmembrane ubiquitin ligase complex ZNRF3/RNF43. Inactivation of the ZNRF3/RNF43 complex protects Frizzled GPCRs on the cell surface from degradation, potentiating Wnt signaling (Doherty and Sanjay, 2020). Loss of LGR4 promotes BMSC proliferation but inhibits osteogenic differentiation and *in vivo* bone mass accrual in mice (Luo et al., 2009; Sun et al., 2019). Similarly, LGR5 knockdown suppresses osteogenesis, in part through impaired mitochondrial activity and reduced expression of β-catenin (Lin et al., 2019), while overexpression of LGR5 in pre-osteoblastic MC3T3-E1 cells enhances osteoblast differentiation (Yu et al., 2021). Finally, multiple studies report significant LGR6 expression in osteoblast progenitors (Khedgikar and Lehoczky, 2019; Liu et al., 2019). LGR6 is strongly induced throughout osteogenic differentiation of BMSCs, compared to only modest increases in LGR4 (Khedgikar and Lehoczky, 2019). LGR6 knockdown in mice reduces trabecular bone mass, inhibits skeletal stem cell self-renewal, and impairs bone regeneration (Doherty et al., 2023; Khedgikar et al., 2022). Remarkably, LGR6-dependent enhancement of osteogenesis occurs through both Wnt-dependent and Wnt-independent signaling cascades, demonstrating redundancy in promoting BMSC osteogenic commitment (King et al., 2024). Through R-spondin agonism, LGRs may represent significant future bone anabolic therapeutic targets.

### 6.3 Adhesion GPCRs

Initially identified as a potential causal genetic driver of BMD in humans (Sabik et al., 2020), adhesion G protein-coupled receptor 133 (GPR133) has recently emerged as a therapeutic target for enhancing osteogenesis. Activation of GPR133 typically depends on exposure of an internal agonistic *Satchel* sequence via mechanical force (Liebscher et al., 2014; Ping et al., 2022; Stephan et al., 2022) or interaction with its extracellular ligands PTK7 (Frenster et al., 2023) and Plxdc2 (Bianchi et al., 2021). Mice with a germline deletion for GPR133 have reduced cortical thickness, trabecular number, bone volume fraction, and BMD compared to wildtype mice (Lehmann et al., 2025). Osteoblast precursor-specific GPR133 knockout mice exhibited similar reductions in bone mass, as well as reductions in markers of bone formation and osteoblast activity. AP503 was recently identified as the first specific, highly potent small-molecule GPR133 agonist (Yang et al., 2025). Activation of GPR133 via AP503 enhanced bone formation *in vivo* and abrogated bone loss in a mouse ovariectomy model (Lehmann et al., 2025). A separate study recapitulated these results and established that GPR133 activation via GL64, another small molecule agonist, also inhibits osteoclastogenesis through the cAMP-PKA-NFATC1 pathway (He et al., 2025).

A closely related adhesion GPCR, GPR126, also regulates skeletal development (Liu et al., 2021; Sun et al., 2020; Bian et al., 2024). Deletion of GPR126 in osteoblasts delays bone formation and mineralization in embryonic mice, as evidenced by reductions in osteocalcin and Col1a1 expression (Sun et al., 2020). Similar reductions in bone length and bone volume are evident with chondrocyte-directed GPR126 knockdown (Bian et al., 2024). Postnatal reductions in bone volume, BMD, trabecular thickness, and bone strength are also observed, with poor osteoblast proliferation, differentiation, and mineralization. Intriguingly, osteoblasts lacking GPR126 demonstrate reduced levels of intracellular cAMP and phosphorylated CREB, which are alleviated by teriparatide treatment (Sun et al., 2020). Therefore, GPR126 regulation of the cAMP-CREB pathway is required for proper osteogenesis, underscoring the therapeutic potential of this receptor.

### 6.4 CB1 and CB2

Cannabinoid receptors 1 (CB1) and 2 (CB2) signal through the inhibitory G_i_ and G_o_ proteins, and their activation leads to inhibition of adenylyl cyclase, activation of MAPKs, inhibition of certain voltage-gated calcium channels, and activation of GIRKs, though ion channel modulation by CB2 is more variable than CB1 (Howlett et al., 2002). CB1 is abundantly expressed in the central nervous system (Mackie, 2005), while CB2 is expressed primarily in peripheral organs with immune function (Brown et al., 2002; Galiègue et al., 1995; Munro et al., 1993). CB1 and CB2 also regulate bone homeostasis. CB1 exerts age-dependent effects on bone mass through modulation of both osteoblast and osteoclast activity, with genetic inactivation of CB1 resulting in higher bone mass in young mice (Bab, 2007; Bab and Zimmer, 2008; Idris et al., 2005). However, CB1-deficient mice still develop age-related osteoporosis due to adipocyte accumulation in the bone marrow (Idris et al., 2005). Separate reports show that CB1 activation promotes corticosteroid-induced osteoporosis in young rats but prevents it in aged rats (Samir and Malek, 2014). CB1 inverse agonists AM251 and SR141716A also induce osteoclast apoptosis and reduce their differentiation (Idris et al., 2008; Idris and Ralston, 2012).

There is a strong correlation between CB2 polymorphisms and women with postmenopausal osteoporosis (Karsak et al., 2005). BMSCs from osteoporotic patients have reduced CB2 expression and restoration of CB2 improves osteogenesis *in vitro* (Wang B. et al., 2018). CB2-deficient mice have fewer osteoblast precursor cells with concurrent increases in osteoclast number and activity, thus resulting in reduced bone mass that worsens with age (Ofek et al., 2006). CB2 agonism with HU308 also prevents ovariectomy-induced bone loss by stimulating osteogenesis (Sophocleous et al., 2011). Another CB2 agonist (2-arachidonylglycerol (2-AG)) similarly restores ovariectomy-induced reductions in bone density and microarchitecture. In human BMSCs, 2-AG also enhances proliferation and osteogenic differentiation (Tian et al., 2021). Finally, CB2 agonists may also protect against cancer-induced bone loss in metastatic settings (Lozano-Ondoua et al., 2010). However, targeting CB2 for osteoporosis is challenging because this receptor also regulates neurological and immune functions (Jourdan et al., 2017).

### 6.5 GLP-1R

The primary physiological role of glucagon-like peptide-1 (GLP-1) is to regulate glucose levels by stimulating insulin secretion and inhibiting glucagon secretion (Hare et al., 2009). While the class B GPCR GLP-1 receptor (GLP-1R) was first identified in pancreatic islet β-cells and the central nervous system (Drucker et al., 1987; Shimizu et al., 1987), it is also expressed in osteoblasts and osteoclasts (Koren et al., 2014; Yamada et al., 2008). The role of GLP-1R in regulating bone strength is controversial. Young mice lacking GLP-1R have reduced tibial and vertebral cortical bone volume and strength compared to wildtype littermates (Yamada et al., 2008). However, no significant change in bone mineral quantity or quality was reported in a separate study using GLP-1R knockout mice, though they displayed reduced cortical layer thickness, bone diameter, bone mineral content, and strength due to a significantly immature collagen matrix (Mabilleau et al., 2013).

GLP-1R agonists and their analogs alleviate diabetes-induced osteoporosis (Mabilleau et al., 2018; Cheng et al., 2022). The GLP-1R agonist Exendin-4 (exenatide) enhances osteoblast proliferation and ALP activity *in vitro* (Zhang et al., 2019), and induces bone formation *in vivo* (Ma et al., 2013; Nuche-Berenguer et al., 2009; Nuche-Berenguer et al., 2010; Nuche-Berenguer et al., 2011; Wang et al., 2017). Numerous *in vitro* and *in vivo* studies have also demonstrated that liraglutide, another GLP-1R agonist, promotes bone formation while inhibiting bone resorption (Cheng et al., 2022; Li et al., 2020; Lu et al., 2015; Sun et al., 2015; Wen et al., 2018; Wu et al., 2017; Yang et al., 2019). Finally, the agonist semaglutide enhances bone mass in ovariectomized rats (Abo-Elenin et al., 2025) and stimulates BMSC proliferation (Tian et al., 2025).

While these *in vitro* and rodent study results are promising, results from human studies have been inconsistent. Examination of BMD and bone turnover markers in Type 2 Diabetes Mellitus patients treated with liraglutide and exenatide found that the GLP-1R agonists had no effect (Gilbert et al., 2016; Li et al., 2015b). While one meta-analysis revealed that liraglutide reduced fracture risk, the opposite was true for exenatide (Mabilleau et al., 2016; Sun et al., 2015). Given these inconsistencies, further research is needed before considering GLP-1R agonism as a therapeutic for low bone mass.

### 6.6 Fatty acid-binding GPCRs

As evidenced with GLP-1R, the relationship between metabolism, body mass, and bone health is complex. Obesity is correlated with increased bone mass due to enhanced mechanical loading (Zhao et al., 2007). However, obesity is also characterized by chronic inflammation, insulin resistance, and dyslipidemia, all of which have independent negative effects on bone (Rinonapoli et al., 2021). The effects of dietary components and metabolism on bone are equally complicated, but there is compelling evidence that fatty acid-binding GPCRs directly regulate skeletal turnover (Kim et al., 2026). GPR120 (free fatty acid receptor 4; FFAR4) receptors are activated by medium- and long-chain fatty acids, integrating metabolic signals into cellular responses. In an ovariectomy model, transgenic mice that convert endogenous omega-6 fatty acids into omega-3 fatty acids have higher BMD, trabecular bone volume, and trabecular number compared to wildtype littermates, effects that are mediated by signaling through the fatty acid-binding GPCR GPR120. Subsequent treatment with docosahexaenoic acid (DHA), an omega-3 fatty acid, stimulates osteoblast viability and differentiation through the Wnt/β-catenin pathway, while osteoclastogenesis is suppressed via NF-κB inhibition (Ahn et al., 2016). Administration of high-dose TUG-891, a potent activator of GPR120, also attenuates ovariectomy-induced bone loss in mice, alongside promoting BMSC differentiation into osteoblasts (Gao et al., 2015).

GPR120 is not the only fatty acid-binding GPCR that regulates bone formation. Stimulation of GPR40 with the agonist GW9508 stimulates early markers of differentiation in murine BMSCs and pre-osteoblast MC3T3-E1 cells but ultimately inhibits mineralization late in differentiation (Philippe et al., 2016). By contrast, treating pre-osteoblastic MC3T3-E1 cells with the Bile acid receptor 1/Takeda G-protein coupled receptor 5 (TGR5) agonist GPBARA elevates osteogenic marker gene expression and mineralization via the AMPK pathway, which is attenuated upon siRNA-mediated knockdown of TGR5 (Wang Q. et al., 2018). Dual targeting of TGR5 and Farnesoid X receptor (FXR), a master regulator of bile acid metabolism, markedly improves bone mass in ovariectomy and aging models, although these effects are primarily mediated by reduced activity of osteoclasts (Li et al., 2019). Taken together, GPCRs associated with fatty acid metabolism offer new, and potentially non-pharmacologic, strategies for bone mass acquisition.

### 6.7 Neuropeptide-activated GPCRs

Neuropeptide GPCR signaling axes play a significant role in balancing bone metabolism (Mills et al., 2024; Lin et al., 2025). Antagonists of the GPCR neuropeptide Y1 receptor (NPY1R) induce osteogenic differentiation of BMSCs and attenuate ovariectomy-induced bone loss in rats, while NPY1R overexpression inhibits BMSC differentiation (Liu et al., 2016; Yu et al., 2016; Xie et al., 2020a). By contrast, Neuropeptide VF precursor (NPVF), which binds to the neuropeptide FF GPCRs NPFFR1/GPR147 and NPFFR2/GPR74, enhances osteogenic differentiation *in vitro* and *in vivo*. NPVF-treated BMSCs exhibit increased mineralization and ALP activity via upregulation of the Wnt/β-catenin pathway, which is abrogated by the NPFFR1 antagonist RF9 and Wnt antagonist JW74. Scaffolds coated with nanofiber CsgA-NPVF also enhance bone formation in a rat calvarial defect model, highlighting the therapeutic potential of NPVF in skeletal repair (Yu et al., 2023). Interestingly, the effects of NPVF on the skeleton *in vivo* are sex-specific, as only female *Npvf* ^*−/−*^ mice show enhanced femur mineral content compared to WT controls which is reversed upon feeding with a high fat diet (Herzog et al., 2026).

Multiple studies have demonstrated a positive role of neuropeptide kisspeptin, which binds the GPCR KISS1R/GPR54, on bone formation. Acute administration of kisspeptin (90 min) enhances circulating osteocalcin levels in young adult men. Kisspeptin treatment of human BMSCs also increases ALP, while robustly inhibiting osteoclastogenesis (Comninos et al., 2022). Complementary murine studies show that a smaller, short-acting kisspeptin fragment, kisspeptin-10 (KP-10) dose- and time-dependently induces the expression of osteogenic transcription factors Distal-less homeobox 5 (Dlx5) and Runt-related transcription factor 2 (Runx2) in BMSCs. Furthermore, KP-10 induces BMP2 expression and Smad phosphorylation via the NFATc4 pathway in C3H10T1/2 cells, thus promoting BMSC osteogenic differentiation (Son et al., 2018). Collectively, these studies establish various neuropeptide-mediated GPCR signaling cascades in the regulation of BMSC activity, with kisspeptin serving as a key example of a therapeutically translatable neuropeptide target.

### 6.8 GIRKs

New therapeutic targets may also arise from examining effectors of GPCR signaling. G protein-gated inwardly rectifying K^+^ (GIRK) channels are part of the Kir family of inward rectifiers and are expressed in multiple tissues, including the nervous system, heart, pancreas, and musculoskeletal tissue (Bond et al., 1995; Ferrer et al., 1995; Kurachi et al., 1986; Nguyen et al., 2024; Taylor et al., 2022). Mammals express a total of four GIRK subunits (GIRK1-4) that combine to form homo- or heterotetrameric functional complexes (Jeremic et al., 2021; Lüscher and Slesinger, 2010; Nguyen et al., 2024). Various ligands, such as acetylcholine, dopamine, opioids, serotonin, somatostatin, adenosine, and GABA, stimulate their cognate GPCRs and couple specifically to pertussis toxin (PTX) sensitive heterotrimeric G proteins, resulting in GIRK activation (Lüscher and Slesinger, 2010; Pfaffinger et al., 1985).

Due to their known roles in excitable cells, GIRK channels are best characterized in the central nervous system and heart (Campos-Ríos et al., 2022; Luo et al., 2022). GIRK channel modulation also affects cells of the mesenchymal lineage, regulating adiposity and energy homeostasis (González-García and Xu, 2025; Oh et al., 2023; Perry et al., 2008), as well as BMSCs. Of the four GIRK subunits, GIRK3 has shown promise as a therapeutic target for bone anabolism. Mice with a germline deletion for GIRK3 have longer femora and tibiae (Taylor et al., 2022). Additionally, Girk3^−/−^ mice have elevated bone mass that is acquired with age and dependent, at least in part, on Wnt signaling (Weaver et al., 2024). Girk3^−/−^ BMSCs are also more proliferative and osteogenic than those of their wildtype littermates. The main effects of GIRK3 deletion are on the mesenchymal lineage in bone, as GIRK3 knockdown in osteoblast precursors recapitulated, while monocyte-directed deletion of GIRK3 did not recapitulate, high bone mass phenotypes of Girk3^−/−^ animals (Weaver et al., 2024). Taken together, GIRK3 inhibits BMSC proliferation and differentiation as well as bone formation. However, germline deletion of a specific gene is not a viable therapeutic strategy, thus necessitating the use of pharmacological modulators.

Pharmacological modulation of GIRK channels to either induce or inhibit their activation is feasible and an area of rapid discovery. ML297 is a potent GIRK1/2 channel activator, though it also has a weak effect on GIRK1/3 and GIRK1/4 channels (Kaufmann et al., 2013; Sánchez-Rodríguez et al., 2020). Another activator, VU0529331, was first compound developed to preferentially activate non-GIRK1/X channels, and it also has potential as a template for even more selective activators (Kozek et al., 2019). Tertiapin-Q (TQ), a synthetic derivative of a natural peptide found in honeybee venom, potently inhibits GIRK1/4 and to a lesser extent GIRK1/2 channels in a dose-dependent manner, although it has been reported that a small percentage of GIRK channels remain unaffected (Jin and Lu, 1998; Jin and Lu, 1999; Montalbano et al., 2015; Walsh, 2011). Despite the emerging evidence regarding the therapeutic potential of GIRK3, there are no GIRK3-specific pharmacological modulators currently available. As this represents a major knowledge gap, future studies should focus on the development of a GIRK3-specific inhibitor that recapitulates the bone and other phenotypes observed in GIRK3 null mice.

### 6.9 GRKs

Expressed in several cell subtypes within the bone marrow niche, GRKs negatively regulate GPCR signaling (Brozowski et al., 2014; Lefkowitz, 1998). This inhibition is achieved by GRK-mediated phosphorylation of an active GPCR followed by binding of either arrestin or β-arrestin, which sterically inhibits signal transduction between the receptor and the G protein (Bliziotes et al., 2000; Lefkowitz, 1998). To date, a total of seven GRK isoforms (GRK1-7) have been identified (Pitcher et al., 1998), with GRK2 and GRK3 most relevant in bone (Wang et al., 2004). GRK2 regulates PTH1R signaling in osteoblasts, as mice with osteoblast-directed GRK2 inhibition have enhanced bone remodeling and PTH1R-stimulated cAMP accumulation (Flannery and Spurney, 2001; Spurney et al., 2002; Spurney, 2003). Low concentrations of tumor necrosis factor alpha (TNF-α) also inhibit GRK2 activity, similarly enhancing osteogenesis (Daniele et al., 2017). Conversely, osteoblast-specific overexpression of GRK2 promotes bone loss by attenuating PTH-induced cAMP generation (Wang et al., 2005). While these results suggest GRK2 inhibition as a promising therapeutic strategy, osteoblastic expression of the dominant negative mutant GRK2 (K220R) inhibits osteoblast proliferation (Bliziotes et al., 2000). More recent research has shown that GRK3-deficient BMSCs also exhibit enhanced proliferation and undergo rapid osteogenic differentiation *in vitro* (Brozowski et al., 2022). Despite this, there are no observable differences in bone volume fraction nor microarchitecture in GRK3-deficient mice (Brozowski et al., 2022). Similarly, although GRK3 deficiency enhances osteoclastogenesis *in vitro* and proliferation of osteoclast precursors *in vivo*, osteoclast-mediated bone resorption is unaffected (Rabjohns et al., 2023).

## 7 Conclusions and future directions

Osteoporosis is a debilitating, multifactorial disease that affects individuals across the lifespan. The overlapping and divergent roles of GPCR signaling in each step of osteogenesis, from BMSC proliferation to fate determination and survival permits fine-tuning of their application as anabolic therapeutics. As of early 2026, there are over 50 clinical trials registered on clinicaltrials.gov focused on osteoporosis and BMSCs. From optimizing delivery methods to oral and long-acting formulations, expanding indications for sclerostin inhibitors, and exploring further antibody-based therapies, the current landscape of clinical trials is rich with paradigm-shifting strategies to protect skeletal health.

GPCRs are widely distributed throughout the mammalian body and regulate a broad array of physiological processes. Therefore, opportunities for “new” bone anabolic therapies may lie in repurposing GPCR-mediated drugs with other indications. For example, β1-selective blockers, which were originally designed to manage cardiovascular conditions, also enhance BMD (Khosla et al., 2018). Similarly, randomized controlled trials in postmenopausal women with osteopenia taking melatonin, which signals through the class A MT1 and MT2 GPCRs, also have elevated BMD (Amstrup et al., 2015). Repurposing of the current library of GPCR-mediated therapeutics may uncover previously unrecognized skeletal benefits.

The fact that osteoporosis is a comorbid condition makes disentangling these benefits, or side effects, a challenge. Fortunately, cutting-edge preclinical *in vitro* and animal models are still actively seeking to define the basic mechanisms underlying bone remodeling and metabolism. Continued integration of mechanistic GPCR biology with human clinical investigation will be essential to fully realize the next-generation of targeted bone anabolic therapies.
